# Phage and endolysin-based inhibition of *Klebsiella pneumoniae*: from mechanisms to combination therapies

**DOI:** 10.3389/fcimb.2026.1819965

**Published:** 2026-04-29

**Authors:** Yaojia Xia, Zhu Chen, Jieting Pan, Xibang Liu, Lijian Ding, Liming Jiang

**Affiliations:** 1 Department of Respiratory and Critical Care Medicine, The Affiliated Yangming Hospital of Ningbo University (Yuyao People’s Hospital), Ningbo University, Ningbo, Zhejiang, China; 2 Ningbo Key Laboratory of Malignant Tumor Immunodiagnosis and Immunotherapy, Health Science Center, Ningbo University, Ningbo, Zhejiang, China; 3 School of Basic Medical Sciences, Health Science Center, Ningbo University, Ningbo, Zhejiang, China; 4 Department of Clinical Laboratory Medicine, Ningbo No.2 Hospital, Wenzhou Medical University, Ningbo, Zhejiang, China; 5 School of Pharmacy, Health Science Center, Ningbo University, Ningbo, Zhejiang, China

**Keywords:** combination therapy, depolymerase, endolysin, *Klebsiella pneumoniae*, phage therapy, phage training

## Abstract

*Klebsiella pneumoniae* (*K. pneumoniae*) is a critical ESKAPE pathogen with rapidly escalating resistance to last−line antibiotics, for which phages and their derived endolysins have emerged as promising alternatives. Phages specifically recognize bacterial surface structures, including capsular polysaccharide (CPS) and lipopolysaccharide (LPS), with their depolymerases degrading these barriers and disrupting biofilms to attenuate virulence. In parallel, endolysins directly cleave the peptidoglycan layer to induce rapid bacterial lysis. Animal model studies confirm that both phages and endolysins effectively reduce *K. pneumoniae* burden and improve host survival, with efficacy markedly enhanced when combined with antibiotics or antimicrobial peptides (AMPs). Although narrow host range and potential resistance evolution remain challenges, strategies such as phage cocktails, “phage training”, and combination therapies (e.g., phage-antibiotic, phage-AMP, and endolysin-antibiotic combinations) have shown broad application prospects. This review details the antibacterial actions of phages and endolysins against *K. pneumoniae*, including their mechanisms, anti−biofilm activity, and therapeutic potential in combination therapies.

## 1 Introduction

*K. pneumoniae* is a Gram-negative bacillus belonging to the *Enterobacteriaceae* family, widely distributed in the natural environment and the human gastrointestinal tract (Noreika et al., 2023). As a key member of the ESKAPE group of drug-resistant bacteria, *K. pneumoniae* is a clinically important opportunistic pathogen that accounts for approximately 30% of all Gram-negative bacterial infections (Paczosa and Mecsas, 2016; Li et al., 2023). It can cause various diseases, including pulmonary infections, urinary tract infections, and bacteremia. The mortality rate of *K. pneumoniae*-induced bacteremia ranges from 16% to 40%, underscoring its significant clinical threat (Tan et al., 2024).

In terms of the hazards of drug resistance, carbapenem-resistant *K. pneumoniae* (CRKP) causes over 90,000 infections and more than 7,000 deaths annually in Europe, which accounts for 25% of mortality cases related to multidrug-resistant (MDR) bacterial infections (Cassini et al., 2019). Data from the U.S. Centers for Disease Control and Prevention (CDC) showed that among 9,000 infections caused by carbapenem-resistant Enterobacterales (CRE) in 2013, 80% were attributed to antimicrobial-resistant (AMR) *K. pneumoniae* (Solomon and Oliver, 2014). A 2019 global systematic analysis of antimicrobial resistance further revealed that AMR *K. pneumoniae* directly caused over 150,000 deaths and contributed to more than 600,000 total deaths (Ranjbar and AM, 2022).

*K. pneumoniae* has two major pathotypes: the hypervirulent *K. pneumoniae* (hvKP) and the classical *K. pneumoniae* (cKP). HvKP primarily infects healthy individuals and causes community-acquired infections, while cKP is a major nosocomial pathogen that accounts for 10% of all hospital-acquired infections and predominantly infects immunocompromised populations (Farhadi et al., 2021). *K. pneumoniae* produces multiple virulence factors (e.g., CPS, LPS, siderophores) to promote its colonization, survival, and transmission in the host (Tan et al., 2019). The capsule is one of its key virulence factors. Among the various capsular serotypes, K1 and K2 are most closely associated with hvKP and pose a significant threat to human health, particularly in China and other Asian countries (Han et al., 2023).

Antibiotics have long been the mainstay for treating bacterial infectious diseases. However, the irrational use of antibiotics has led to severe bacterial drug resistance. It is projected that by 2050, antibiotic-resistant infections will become the leading cause of global mortality, thereby posing a major threat to public health (Rotman et al., 2024). Even in countries like China that have implemented antimicrobial stewardship and surveillance policies, the detection rate of common drug-resistant bacteria continues to rise (Luo et al., 2024). Moreover, new resistance mechanisms are constantly emerging. Thus, the overall situation of antimicrobial resistance remains grim.

Data from the China Antimicrobial Surveillance Network (CHINET) showed that in 2022, the resistance rates of *K. pneumoniae* to β-lactam antibiotics (cefotaxime and piperacillin-tazobactam) were respectively 42.7% and 26.6% (Qin et al., 2024). The resistance rate of *K. pneumoniae* to meropenem increased markedly from 2.9% in 2005 to 30.0% in 2023 (Hu et al., 2024). Additionally, CRKP resistance to colistin rose from 3.6% in 2020 to 8.2% in 2022, while resistance to fosfomycin remained stable at approximately 50% between 2019 and 2022. Due to *K. pneumoniae*’s high resistance to last-resort antibiotics, including third-generation cephalosporins and carbapenems, the World Health Organization (WHO) has listed it as a critical priority pathogen in both its 2017 and 2024 reports (Mirza et al., 2025). These findings underscore the worsening drug resistance of *K. pneumoniae*, particularly the spread of MDR *K. pneumoniae*, which poses enormous challenges to clinical treatment.

Phages are viruses that specifically infect and lyse bacteria. They can target and kill specific host bacteria, including antibiotic-resistant isolates, thus making them promising alternatives to antibiotics for treating *K. pneumoniae* infections. Compared with antibiotics, phages exhibit self-amplification, strong host specificity, and low toxicity. They not only minimize disruption of the normal microbiota as well as degrade established biofilms, but also have an extremely low probability of cross-resistance with antibiotics (Patil et al., 2021; Chang et al., 2022). Additionally, phages offer the advantages of low cost and high safety. Their antibacterial mechanism primarily involves specifically recognizing *K. pneumoniae*, invading bacterial cells to complete replication, and ultimately causing bacterial lysis and death (Dunstan et al., 2021). Furthermore, studies have confirmed synergistic interactions between phages, antibiotics, and the host immune response. Phages can degrade bacterial capsules or biofilms, thereby creating favorable conditions for antibiotics, antibodies, the complement system, and phagocytes to exert their effects (Mohammadi et al., 2023). This finding further suggests the powerful therapeutic potential of combining phages with antibiotics, AMPs, and other agents.

Given the growing interest in phage therapy, research has expanded to explore phage-encoded enzymes (e.g., endolysins and polysaccharide depolymerases) as novel antibacterial agents. Compared with phages, phage-encoded enzymes that do not directly target bacteria are less likely to induce bacterial resistance. These enzymes lack replication ability, have a broader host range while maintaining high host specificity, and pose a low risk of inducing bacterial resistance. Additionally, their regulatory pathways are simpler than those of whole phages, thus offering higher accessibility and bioavailability (Zhang et al., 2025). Phage endolysins are peptidoglycan hydrolases expressed by phages in the late stage of bacterial infection. They lyse bacterial cell walls, which leads to bacterial death and the release of progeny phages (Antonova et al., 2019).

In this review, we summarize the mechanisms of *K. pneumoniae* resistance to major classes of antibiotics (e.g., enzymatic inactivation/modification of antibiotics, alteration of antibiotic targets, porin loss, and mutation). We also provide an overview of *K. pneumoniae* phages and their research in animal models, with a focus on the mechanisms of action of phages and their endolysins in treating *K. pneumoniae* infections (e.g., attenuation of *K. pneumoniae* virulence, antibiofilm activity, phenotypic modification of bacteria). Furthermore, we discuss the research progress and prospects of emerging therapeutic strategies such as “phage training” and combination antibacterial therapy in the era of widespread drug resistance.

## 2 Mechanisms of *K. pneumoniae* resistance to major antibiotic classes

**Figure 1:**
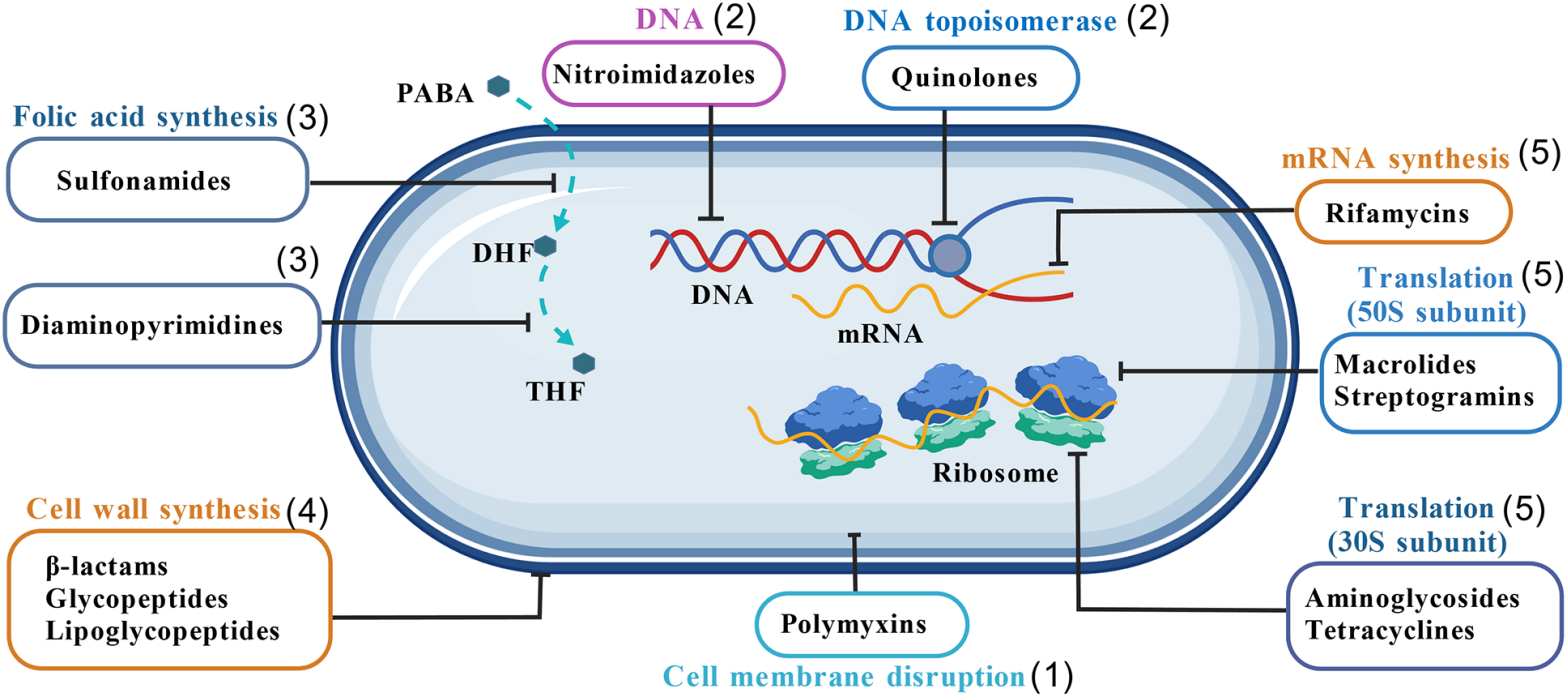
Mechanisms of action of antibiotics: (1) cell membrane disruption: polymyxins and Lipopeptides disrupt the structure and function of the bacterial cell membrane, leading to leakage of cellular contents and bacterial death. (2) Inhibition of DNA-related processes: Nitroimidazoles and Nitrofurans interfere with DNA synthesis or function; Quinolones target DNA topoisomerases, inhibiting replication and transcription. (3) Inhibition of folic acid synthesis: Diaminopyrimidines and Sulfonamides block different stages of folic acid synthesis, which is essential for bacterial growth. (4) Inhibition of bacterial cell wall synthesis: β-Lactams, Glycopeptides, and Lipoglycopeptides inhibit cell wall formation, causing cell lysis and death, particularly in actively growing bacteria. (5) Inhibition of protein synthesis: Rifamycins suppress mRNA synthesis; Macrolides and others act on the 50S ribosomal subunit, while Aminoglycosides and Tetracyclines target the 30S subunit, disrupting protein synthesis and bacterial growth.

Understanding the mechanisms of antibiotic action is critical for comprehending the development of bacterial resistance. In general, antibiotics exert their effects through five primary mechanisms: interfering with peptidoglycan biosynthesis, nucleic acid synthesis, protein synthesis, the function of metabolic enzymes, and disrupting the cytoplasmic membrane (Pulingam et al., 2022). As antibiotics may act through one or more of these mechanisms, their modes of action are summarized in **Figure 1**.

**Figure 2:**
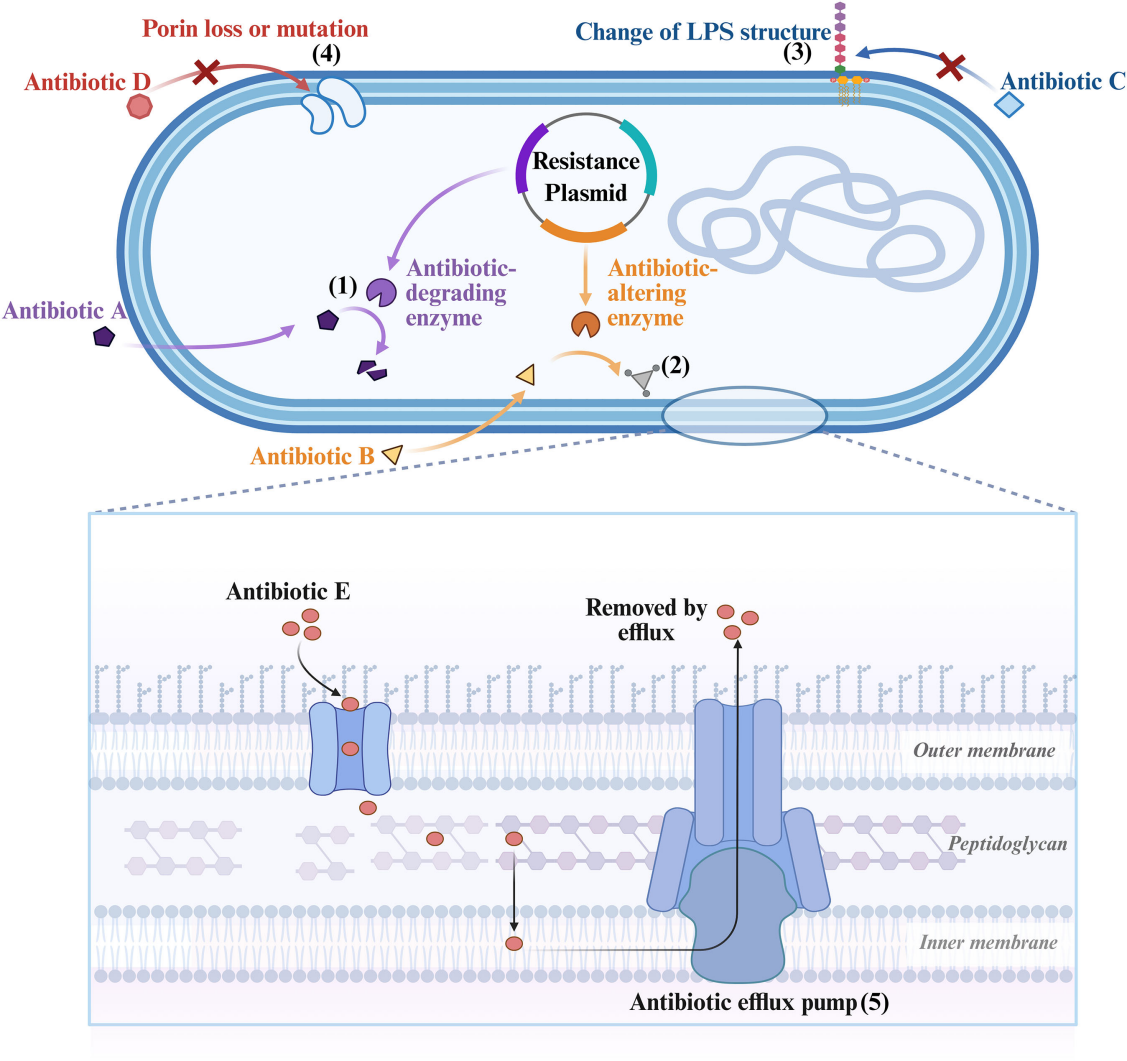
Antibiotic resistance mechanisms in *K. pneumoniae*: (1) enzymatic degradation: antibiotic-degrading enzymes, such as β-lactamases, hydrolyze and inactivate the antibiotic molecules. (2) Enzymatic alteration: Antibiotic-altering enzymes modify the drug’s structure, a mechanism that reduces its binding affinity for the target. (3) Target site alteration: Mutations that alter the structure of LPS or other cell wall components can prevent antibiotic binding. (4) Reduced permeability: Modifications that lead to the loss or mutation of outer membrane porins restrict antibiotic entry into the cell. (5) Active efflux: Antibiotic efflux pumps expel the drug from the cell, thereby reducing the intracellular concentration to a level that is below the effective threshold.

*K. pneumoniae* has evolved multiple strategies to counteract the effects of antibiotics. These strategies are summarized in **Table 1**; **Figure 2**. They include enzymatic inactivation of drugs, alteration of target sites, reduced drug uptake due to porin loss or mutation, active efflux via overexpression of efflux pumps, and biofilm formation (Li et al., 2023). However, resistance rarely arises from a single pathway. Instead, it often results from the synergy of multiple mechanisms, including the role of mobile genetic elements in driving the rapid spread of resistance genes (Zdarska et al., 2026). Mobile genetic elements such as plasmids, prophages, transposons, and insertion sequences mediate the horizontal transfer of antibiotic resistance genes and virulence determinants. This process has led to the emergence of carbapenem-resistant hypervirulent strains, a particularly concerning threat (Han et al., 2025; Pan and Li, 2025). In addition to these stable genetic changes, adaptive programs can also shape transient tolerance. These include stress responses that modulate gene expression under antibiotic pressure, as well as the formation of persister cells that survive antibiotic exposure without genetic changes (Zdarska et al., 2026).

**Table 1:** Resistance mechanisms of *K. pneumoniae* to major antibiotic classes.

Antibiotic category	Representative drugs	Mechanism of drug resistance generation	Reference
Cephalosporins	CAZ-AVI, Cefepime	Porin alterations and gene mutations	(Le et al., 2023)
Polymyxins	Polymyxin B, E	Chromosomal mutations (*pmrCAB*, *phoPQ*)/Plasmid *mcr* genes	(Han et al., 2023)
Tetracyclines	Tigecycline (TGC), Doxycycline, Minocycline	Enzyme-mediated inactivation (AMEs, methyltransferases)	(Maxwell, 2016)
Aminoglycosides	Streptomycin, Gentamicin, Amikacin	Enzyme-mediated inactivation (AMEs, methyltransferases)	(Hung et al., 2011)
Phosphorus-rich compounds	Fosfomycin	Transporter defects/Target modification/FosA enzyme	(Assafiri et al., 2021)
Carbapenems	Imipenem, Meropenem, Panipenem, Ertapenem	Carbapenemase acquisition (*bla*_KPC_, *bla*_OXA-48_, *bla*_NDM-1_, *bla*_IMP-1_, etc.)	(Liu et al., 2023)
Fluoroquinolones	Norfloxacin, Ciprofloxacin, Ofloxacin, Levofloxacin	Target gene mutations (*parC*, *gyrA*)	(Denissen et al., 2022)

Beyond the above mechanisms, the quorum sensing system regulates group behaviors including biofilm formation, virulence factor production, and antibiotic resistance through the upregulation of efflux pump expression (Venkatesan et al., 2025). Recent transcriptomic analysis has shown that upon meropenem exposure, *K. pneumoniae* rapidly activates adaptive stress responses. This activation involves a more pronounced upregulation of biofilm-related genes, efflux pump-related genes, and membrane remodeling-related genes than β-lactamase genes (Carstensen et al., 2025). These findings highlight quorum sensing as a promising target for anti-resistance adjuvant therapy. Moreover, resistance has also been documented against newer agents such as ceftazidime-avibactam (CAZ-AVI), imipenem-relebactam, and cefiderocol. For CAZ-AVI, resistance is driven by mutations in *blaKPC* genes that produce KPC variants with reduced avibactam affinity (Fu et al., 2025b; Liu et al., 2025). Cefiderocol resistance involves mutations that downregulate siderophore receptor genes or repress the TonB complex, thereby compromising drug uptake (Pu et al., 2026). Beyond these specific pathways, heteroresistance has emerged as a critical clinical concern. Studies have shown that 97% of clinical *K. pneumoniae* isolates exhibit heteroresistance to at least one antibiotic, and 72% of strains show heteroresistance to two or more agents (Zhang et al., 2025a). This phenomenon often leads to treatment failure because routine susceptibility testing cannot detect resistant subpopulations (Zhang et al., 2025a; Zhang et al., 2025b). Over the past decade, resistance of *K. pneumoniae* to major antibiotic classes has steadily increased, leaving few effective options and driving high mortality (Sati et al., 2025). This alarming trend underscores the urgent need for alternative strategies, such as phage-based therapies, which are the focus of this review.

## 3 *K. pneumoniae* phages

In recent years, AMR in bacterial pathogens has become a major risk factor for the management of infectious diseases. AMR is currently estimated to cause 700,000 deaths globally each year, a figure that is projected to reach 10 million by 2050 (Chang et al., 2022). However, the development of new antibiotics has stagnated due to cost and feasibility issues, thereby creating an urgent need for novel antibacterial therapies.

Phages are the most abundant biological entities on Earth, with an estimated population size of 10³¹ particles. Although the therapeutic potential of phages was first discovered by Felix d’Herelle in the 1910s, their application was sidelined (except in Eastern Europe) following the discovery of penicillin (Chang et al., 2022). Nevertheless, the spread of antibiotic resistance has sparked a revival of interest in phage therapy. The therapeutic use of phages has been extensively studied, and recent clinical successes with personalized phage cocktails have reignited interest in this approach (Hatfull et al., 2022).

Globally, *K. pneumoniae*-targeting phages have been isolated from diverse sources like sewage, rivers, and animal feces. For example, phage ΦKp9438 was found in hospital sewage (Hu et al., 2023). According to the latest ICTV taxonomy update (ratified in 2025), the classification of phages now relies mainly on genomic and evolutionary relationships (Turner et al., 2025). Under this framework, most *K. pneumoniae* phages belong to the class *Caudoviricetes*, which includes tailed double−stranded DNA phages (Adriaenssens et al., 2025). Within this class, different families have been created to reflect the genomic diversity among these phages. For instance, the family *Autographiviridae* includes phages that target K20 and KL64 capsular types (Chen et al., 2025; Zhao et al., 2025). Another family, *Drexlerviridae*, contains phages that infect K1 serotype strains and show good efficacy in animal models (Shi et al., 2026). Also, the family *Demerecviridae* has phages that work against MDR *K. pneumoniae* (Balcão et al., 2022). Overall, these phages vary in shape, genome structure, and infection strategies, which reflects how viral taxonomy continues to evolve with advances in genomics and bioinformatics (Turner et al., 2025). **Table 2** lists representative *K. pneumoniae*-targeting phages under the updated ICTV framework.

**Table 2:** Relevant characteristics of reported representative *K. pneumoniae* phages.

Name	Classification	Genome size	Types of host bacteria	Key enzymes	Antibacterial effect/references
PK2420	*Autographiviridae*	41,155bp	*K. pneumoniae* K20	Unknown	Biofilm inhibition rate: 54–70% (MOI 10² to 10^-^^6^, 48h); *In vitro* growth inhibition: Complete inhibition for ≥4 h (MOI 10² to 10^-^^6^) (Chen et al., 2025)
vB_LSKP32	*Autographiviridae*	40,942bp	*K. pneumoniae* KL64	Unknown	Latent period 5–10 min, burst size ~56 PFU/cell; Elevates *G. mellonella* survival to 90% (MOI 100) vs 15% control (Lan et al., 2026)
vB_KpnM_JYSS3	*Autographiviridae*	47,244bp	*K. pneumoniae* K2	Dep17 (depolymerase)	Latent period 5min (MOI 0.0001); K2-infected mice: blood bacterial load reduced by ≥3.0 Log_10_ CFU/mL (Jiao et al., 2025)
vB_KpP_HS37	*Demerecviridae*	74,084bp	*K. pneumoniae* (clinical isolate)	Lys41 (endolysin)	1.6 Log_10_ CFU/mL reduction on lettuce; 0.9 Log_10_ CFU/mL on cooked chicken (Zhu et al., 2026)
vB_LZ 2044	*Drexlerviridae*	50,419bp	*K. pneumoniae* K1	Unknown	hvKP liver-infected mice: liver bacterial load reduced by ≥4.0 Log_10_ CFU/g (Shi et al., 2026)

Unlike conventional antibiotics, phages exhibit high host specificity and low intrinsic toxicity, properties that minimize disruption to the normal microbiota. A significant advantage of phage therapy is the low likelihood of cross-resistance with antibiotics. Phages can target and kill specific host bacteria, including antibiotic-resistant isolates. Consequently, phages with an exclusively lytic life cycle are preferentially selected for therapeutic applications (Chang et al., 2022). The infection cycle begins when a phage recognizes and binds to specific cell surface structures (e.g., porins, pili, LPS, or CPS) (Cheetham et al., 2024). Following attachment, the phage injects its genetic material into the host bacterium to direct the host’s replication machinery for the production of new virions, ultimately causing bacterial lysis for their release. Different classes of *K. pneumoniae* phages can exert anti-capsular and antibiofilm activity by expressing various polysaccharide depolymerases. Additionally, they mediate bacterial lysis through the action of specific endolysins that degrade the cell wall (Fayez et al., 2023). The host range of *K. pneumoniae* phages varies considerably: some exhibit narrow specificity for particular *K. pneumoniae* strains, while others (e.g., phage LAPAZ) possess a broader tropism and can infect multiple strains (Ziller et al., 2024).

Phage therapy has been successfully deployed to treat various *K. pneumoniae*-induced infections in humans, including intestinal colonization, prosthetic infections, recurrent urinary tract infections, and osteomyelitis (Rotman et al., 2024). Singh et al. reported that a cocktail of phages (ΦKpnBHU4, ΦKpnBHU7, and ΦKpnBHU14) completely eradicated *K. pneumoniae*-induced sepsis (Singh et al., 2022). Manohar et al. demonstrated that phages KPP235, ELP140, and ECP311 achieved 100% survival in *Galleria mellonella* (*G. mellonella*) larvae that had been infected with a mixture of KP235, EL140, and EC311 bacterial strains (Manohar et al., 2018). Furthermore, Gan et al. showed that phages specific for HiAlc *K. pneumoniae* can alleviate alcohol-related liver dysfunction by eliminating this pathogen, thereby altering the gut microbiota (Gan et al., 2023). Despite these promising results, phage therapy is not without limitations. Its inherently narrow host range can inevitably lead to the emergence of phage-resistant bacterial mutants. Yen et al. reported that therapeutic administration of lytic phages in humans was associated with a 28–68% increase in phage resistance genes within the gut microbiota (Yen et al., 2017). This significant challenge has prompted the development of innovative strategies to overcome resistance, including the use of phage cocktails (mixtures of two or more complementary phages), combination therapies with antibiotics, and the application of genetically engineered phages.

## 4 Phage-mediated attenuation of *K. pneumoniae* virulence

The severity of pathogenic infections is critically dependent on both drug resistance and virulence factors (Mohammadi et al., 2023). The emergence of *K. pneumoniae* strains that exhibit high resistance and high virulence has become a global public health crisis. *K. pneumoniae* expresses multiple virulence factors, including CPS, LPS, pili, and siderophores, among which CPS and LPS are particularly critical. The overproduction of CPS confers the hypermucoviscous phenotype, a hallmark of hvKP. At least 80 distinct CPS (K-antigen) types have been reported, among which the K2 serotype is one of the most frequently isolated from prevalent hvKP strains and is strongly associated with severe complications such as liver abscesses, endophthalmitis, and meningitis (Geng et al., 2023).

CPS enables *K. pneumoniae* to resist host phagocytosis and serum killing, while also impeding antibiotic penetration, thereby significantly enhancing bacterial survival against antimicrobial agents (Huang et al., 2024). Additionally, CPS has been demonstrated to protect both prokaryotes and eukaryotes from oxidative stress, a function that has been confirmed in *Streptococcus pneumoniae* and *Cryptococcus neoformans* (Zaragoza et al., 2008; Doyle et al., 2018). LPS is a key component of the outer membrane, consisting of three regions: lipid A, core oligosaccharide, and O-polysaccharide. The O-polysaccharide is instrumental in resisting complement-mediated attack and phagocytosis, thereby allowing the bacteria to evade the host’s innate immune response (Park and Park, 2021). Consequently, the CPS and LPS of *K. pneumoniae* represent core targets for the development of enzyme-based therapeutic strategies. Encouragingly, phage-encoded depolymerases have been shown to disrupt both CPS and LPS of *K. pneumoniae*, thereby demonstrating significant therapeutic potential (Islam et al., 2024).

Phage-encoded depolymerases are multi-domain trimeric proteins localized to tail fibers, baseplates, and neck structures. They recognize and catalyze the degradation of polysaccharides via a central β-helical domain (Zhang et al., 2025). Most phages contain a single depolymerase, while a few (e.g., ΦK64-1) encode up to 11 depolymerases to adapt to multiple capsular types (Pan et al., 2017). Based on their mechanism of action, depolymerases are classified into two major categories: hydrolases and lyases. Hydrolases degrade peptidoglycan, CPS, or the O-antigen side chain of LPS by catalyzing the cleavage of glycosidic oxygen bonds. This category includes sialidases, rhamnosidases, levansucrases, dextranases, xylanases, and LPS deacetylases (Davies and Henrissat, 1995; Stummeyer et al., 2005; Müller et al., 2008; Yee et al., 2011; Ely et al., 2015; Picozzi et al., 2015; Prokhorov et al., 2017). Lyases introduce a double bond between the C5 and C4 of non-reducing uronic acids via β-elimination, a reaction that occurs following the cleavage of the glycosidic bond. This category includes alginate lyases, hyaluronate lyases, pectate lyases, K5 lyases, and O-specific polysaccharide lyases (Jedrzejas, 2000; Scholl et al., 2001; Lindsay et al., 2009; Glonti et al., 2010; Cornelissen et al., 2012; Olszak et al., 2017).

Although depolymerases do not directly kill bacteria, they exert profound effects by degrading surface polysaccharides. Studies have confirmed that depolymerases reduce *K. pneumoniae* virulence by cleaving CPS and can enhance antibacterial efficacy when combined with antibiotics, thereby playing a key role in sensitizing bacteria to both antibiotics and the host immune system. Notable examples of such enzymes include K1 lyase, Depo32, Dp42, depoKP36, KP34gp57, and B1dep (Majkowska-Skrobek et al., 2016; Wu et al., 2019; Pertics et al., 2021; Tu et al., 2022; Cai et al., 2023; Maciejewska et al., 2023). It has been reported that reduced polysaccharide levels facilitate the penetration of antibiotics (particularly positively charged aminoglycosides) through biofilms (Stewart and Costerton, 2001). Furthermore, depolymerases inhibit biofilm formation by degrading the O-polysaccharide of LPS, which enhances the bactericidal effects of other antimicrobials, such as polymyxins, gentamicin, and ciprofloxacin (Verma et al., 2010; Bansal et al., 2015; Wu et al., 2019). For instance, the depolymerase Dep_ZX1 synergizes with gentamicin, streptomycin, and kanamycin, enabling the killing of 99.9% of K57-type *K. pneumoniae* (Li et al., 2022). Moreover, Huang et al. determined the cryo-electron microscopy structure of the depolymerase K64-ORF41, revealing that insertion domains are important evolutionary markers. This finding suggests that depolymerases can evolutionarily adapt to the serotypic diversity of *K. pneumoniae* CPS, thereby highlighting their considerable potential for phage-based biotherapy (Huang et al., 2024).

## 5 Antibiofilm activity of phages

*K. pneumoniae* strains possess a significant capacity for biofilm formation, which is a structured community of bacterial cells enclosed in a self-produced matrix. Biofilms are clusters of microorganisms adherent to surfaces and embedded within an extracellular polymeric substance (EPS), which is a matrix comprising exopolysaccharides, proteins, nucleic acids, and lipids. The composition of this matrix varies depending on the bacterial species, the age of the biofilm, and environmental conditions such as temperature and nutrient availability (Zurabov et al., 2023). It is estimated that over 80% of bacterial infections are associated with biofilms, which typically exhibit heightened resistance to conventional antibiotics (Zhang et al., 2025).

**Figure 3:**
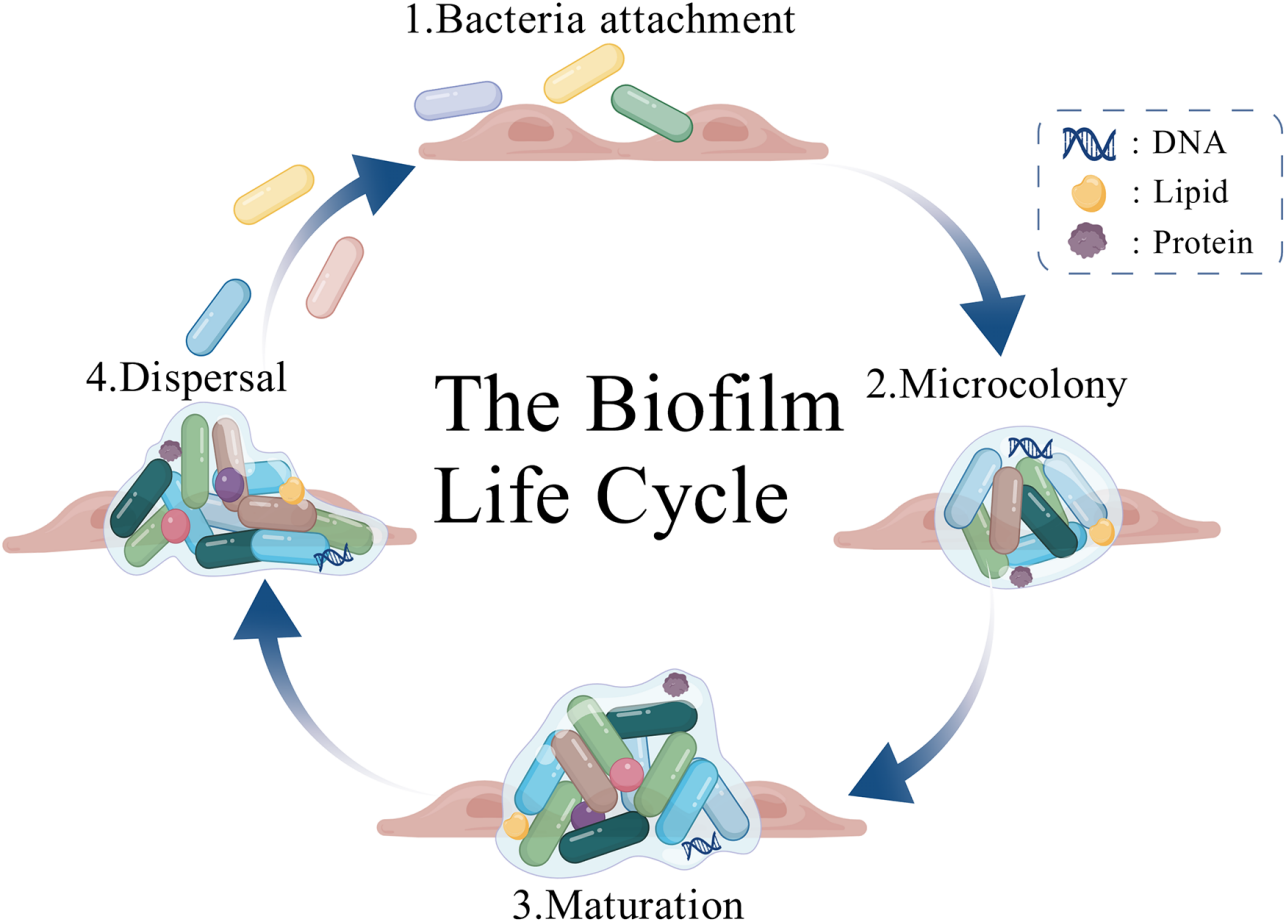
Biofilm formation process: (1) attachment to the surface: bacteria initially adhere to the colonization surface, a process that is mediated by structures such as surface adhesins, fimbriae, and flagella. (2) Microcolony formation: The attached bacteria undergo multiplication, thereby forming microcolonies that become embedded within a self-produced EPS, which includes polysaccharides, proteins, nucleic acids, and lipids. (3) Biofilm maturation: The biofilm structure matures as the secretion of EPS components (polysaccharides, proteins, nucleic acids, and lipids) continues, leading to the development of a complex, three-dimensional architecture. (4) Dispersal and detachment: A subpopulation of bacteria within the microcolonies regulates the expression of proteins associated with motility structures and produces enzymes that degrade the EPS matrix, a key step that facilitates detachment from the surface and initiates the dispersal of cells to new colonization sites.

In human hosts, most biofilms are polymicrobial. The most frequent bacterial partners coexisting with *K. pneumoniae* in mixed communities are *Pseudomonas aeruginosa* (*P. aeruginosa*) and *Proteus mirabilis* (De Los Santos et al., 2024). Within these complex heterogeneous structures, many antimicrobials (including conventional antibiotics) become ineffective due to the physical barrier posed by the EPS and the reduced metabolic activity of the embedded cells (Gilcrease et al., 2023). The process of biofilm formation is delineated in **Figure 3**. Studies have shown that virulence factors (such as CPS, LPS, pili, iron metabolism, and quorum-sensing systems) directly promote *K. pneumoniae* biofilm formation by enhancing initial adhesion and subsequent maturation, thereby considerably increasing treatment difficulty (De Los Santos et al., 2024).

As a potential biocontrol strategy for healthcare facilities to manage MDR *K. pneumoniae* within biofilms, phage therapy offers a distinct advantage: phages can penetrate the aqueous channel network of biofilms and attack/lyse the resident bacteria (Chang et al., 2022). Phages employ diverse mechanisms for antibiofilm activity against *K. pneumoniae*. On one hand, they can disrupt the biofilm architecture by producing extracellular enzymes or through direct adsorption to bacterial surfaces. On the other hand, upon infection, they can encode enzymes such as lysozymes and endolysins, which degrade the bacterial cell wall and induce cell death. Phage-derived depolymerases specifically target and degrade the EPS components of biofilms, an action that disrupts the biofilm structure, reduces bacterial biomass, and promotes deeper phage penetration to enhance bacterial lysis (Chegini et al., 2021). For example, the depolymerase Dep42, encoded by phage SH-KP152226, inhibits and disrupts biofilms of K47-type *K. pneumoniae* (Wu et al., 2019). Conversely, phage KL-2146 can infect *K. pneumoniae* strains carrying the *NDM-1* carbapenemase gene and lyse them by encoding enzymes such as the tail protein H1004_gp67, which disrupts the bacterial cell wall (Gilcrease et al., 2023).

*K. pneumoniae* phages display pronounced diversity in host range and specificity. For instance, the *Siphoviridae* phage vB_KpnS_Kp13 is effective only against specific *K. pneumoniae* isolates that are β-lactamase-producing and members of the ST15 clonal lineage, which express the K24 capsular polysaccharide. This phage has a short latent period and can degrade over 24% and 48% of pre-formed biofilms within 50 and 70 hours, respectively (Horváth et al., 2020). Meanwhile, the *Drexlerviridae* phage KL-2146 exhibits polyvalency, infecting both drug-resistant and susceptible strains. Its genome encodes DNA methyltransferases and depolymerases, which function to evade the host restriction-modification system and degrade the biofilm matrix, respectively (Gilcrease et al., 2023).

*In vitro* and *in vivo* experiments have consistently confirmed that phages significantly inhibit both *K. pneumoniae* growth and biofilm formation. Established methods such as time-kill assays, crystal violet staining, and colony biofilm assays have demonstrated that phages rapidly reduce bacterial counts and disrupt the biofilm’s structural integrity, exhibiting potent antibacterial activity (Sundaramoorthy et al., 2021; Das and Kaledhonkar, 2024; Soontarach et al., 2024). Recent studies have further shown that combining phages with antibiotics, AMPs, and other antimicrobials enhances biofilm clearance. The synergistic mechanisms include disrupting biofilms to facilitate antibiotic penetration, enhancing antibiotic activity, and regulating bacterial metabolism and growth (Talapko and Škrlec, 2020).

## 6 Phage-mediated phenotypic modification of *K. pneumoniae*

Phages play a crucial role in regulating the phenotype of *K. pneumoniae*, thereby affecting its biological characteristics and functions through multiple pathways. Specific phages can directly induce changes in bacterial phenotype and function. For instance, phage ΦFK1979 induces drug resistance in *K. pneumoniae* by altering the colony morphology, CPS expression levels, and related genes in the resulting mutants, thereby reducing their virulence and *in vivo* infectivity (Tang et al., 2023). Furthermore, recombinant proteins encoded by phage SH-KP152410, along with phages KP1, KP12, and depolymerases derived from phage P560, act on *K. pneumoniae* phenotypes by degrading CPS and altering the bacterial survival status (Li et al., 2021; Kim et al., 2022; Li et al., 2022).

Concurrently, phage selection pressure drives adaptive phenotypic changes in drug-resistant *K. pneumoniae*, which manifest as modifications to surface structures, including colony morphology, mucoid production, and capsule thickness. Mutations in CPS biosynthesis-related genes (e.g., *wzc*, *wcaJ*) serve as a key mechanism through which these phenotypic changes are further regulated (Chen et al., 2024). Additionally, studies have shown that engineering endolysins of *K. pneumoniae*-targeting phages not only enhances their antibacterial activity and spectrum but also modifies bacterial surface morphology and increases cell membrane permeability, enabling effective clearance of biofilms and persister cells. This approach shows efficacy in treating mixed bacterial infections (Chen et al., 2024).

## 7 Research on *K. pneumoniae* phages in animal models

As a key preclinical tool, animal models that simulate human bacterial infections are essential for assessing phage therapy efficacy and supporting its clinical development. Mouse and rat infection models are the most widely utilized, whereas large animal models (e.g., rabbits, pigs, sheep, cattle) are less common (Zagaliotis et al., 2022). Additionally, the larvae of *G. mellonella* are extensively employed as preliminary infectious disease models, a preference that is attributable to their ease of handling and the notable similarities between their innate immune system and that of vertebrates. For example, this model has been used to evaluate the antibacterial effects of SAR-phage endolysins against virulent bacteria (Gontijo et al., 2024).

In *K. pneumoniae* infection research, mouse models of pneumonia, peritonitis, wound infection, and urinary tract infection have been established. Results from these models demonstrate that phages effectively reduce the bacterial burden, improve survival rates, and promote wound healing (Kim et al., 2020a; Fayez et al., 2023; Le et al., 2023). Specifically, in a modified mouse tape-stripped skin infection model, the endolysin PlyKp104 was tested for treating *K. pneumoniae*-induced wound infections. This endolysin exhibited intrinsic bactericidal activity in both *in vitro* and *in vivo* assays without requiring membrane permeabilizers or further protein modification (Euler et al., 2023). In a mouse model of systemic infection, injection of the endolysin LysSAP26 conferred protection and improved survival rates, thereby indicating its protective effect against systemic *K. pneumoniae* infections (Kim et al., 2020a). Similarly, in a mouse wound infection model, treatment with phage ZCKP2 reduced the bacterial burden at the wound site and enhanced the healing process (Fayez et al., 2023). Furthermore, in a zebrafish infection model, phages isolated from the Ganges River were shown to inhibit both biofilm formation and planktonic growth of drug-resistant *K. pneumoniae in vitro* (Sundaramoorthy et al., 2021).

While current phage research primarily focuses on therapeutic efficacy, it also encompasses critical areas such as safety, administration routes, pharmacokinetics, and immune responses. Regarding therapeutic efficacy, animal model studies confirm the significant impact of phages on *K. pneumoniae* infections. For example, in zebrafish models, phage KpG effectively inhibits the *in vivo* growth of drug-resistant *K. pneumoniae*, reduces bacterial burden, and exhibits no toxic side effects (Sundaramoorthy et al., 2021). Correspondingly, in mouse models, phage vB_KpnS-Kpn15 significantly reduces *K. pneumoniae* infection rates, improves survival rates, and causes no obvious tissue or organ damage (Zhang et al., 2025).

Safety studies indicate that most phages exhibit no significant toxicity or adverse effects in animals at appropriate doses. However, the impact of phages on the immune system and the potential immune responses they elicit require further thorough investigation. Administration routes including topical application, intraperitoneal injection and intravenous injection have all demonstrated feasibility in animal models. Yet critical challenges remain to be fully addressed, especially in terms of *in vivo* phage stability and immune clearance (Ho et al., 2022). For instance, intravenous injection, while efficient for systemic delivery, suffers from rapid clearance by the innate immune system, with a short half-life of 2.2–4.5 hours in mouse models. This pharmacokinetic obstacle can be mitigated by encapsulation or the selection of long-circulating phage mutants (Dąbrowska, 2019). Regarding immune responses, the innate immune response to *K. pneumoniae* infections has been extensively characterized, whereas studies on the adaptive immune response are relatively limited and predominantly confined to mouse models (Wantuch and Rosen, 2023). For example, Mackel et al. used a C57BL/6 mouse model, which allows dissection of pathogen and host factors to confirm the protective immunity of T cells against *K. pneumoniae* (Mackel et al., 2022). Additionally, selected animal studies on *K. pneumoniae*-targeting phages, used either alone or in combination with antibiotics, are summarized in **Table 3**.

**Table 3:** Animal studies on *K. pneumoniae*-targeting phages alone or in combination with antibiotics.

Animal model	Treatment	Administration route	Key findings	Reference
*G. mellonella* larvae	KP1801 (4×10^6^–4×10^9^ PFU)	Injection	Survival rates (>74-93%), control (50%)	(Wintachai et al., 2020)
Zebrafish larvae	UPM2146 (10^6^ PFU)	Topical exposure	Bacterial load reduction: >7 log_10_ (vs. control)	(Assafiri et al., 2021)
Intraperitoneal infection in mice	vB_KpnS_Kp13 (2×10^8^ PFU)	Intraperitoneal	100% survival in mice	(Horváth et al., 2020)
Bacteremia in mice	Pharr and ϕKpNIH-2 individually or as a cocktail (5×10^7^ PFU)	Intraperitoneal	High survival rate, low phage resistance mutation rate	(Hesse et al., 2021)
Lung infection model in mice	vB_KpnM_P-KP2 (10^7^, 10^8^, or 10^9^ PFU) with/without gentamicin (1.5 mg/kg)	Intranasal	Phage only: >70% survival Combination:100% survival	(Wang et al., 2021)

Beyond animal studies, phages have been successfully applied to treat *K. pneumoniae* infections in humans, including prosthetic joint infections, peritonitis, and urinary tract infections, etc. **Table 4** summarizes representative clinical cases and trials of phage therapy against *K. pneumoniae* in humans.

**Table 4:** Representative clinical cases and trials of phage therapy against *K. pneumoniae* in humans.

Phage/phage cocktail	Patient description	Route	Results	Year/ references
Single phage: KpJH46Φ2	62−year−old diabetic male, prosthetic knee joint infection, amputation offered	Intravenous	Infection resolved, function recovered; asymptomatic at 34−week follow−up; no AEs	2021 (Cano et al., 2021)
Single phage: ΦKp_GWPB35 (first); Cocktail: ΦKp_GWPB35+ΦKp_GWPA139 (second)	Adult inpatient, MDR *K. pneumoniae* pulmonary infection	Nebulization	Phage−resistant mutants emerged (LPS alteration) with reduced virulence; patient clinically improved	2023 (Li et al., 2023)
Cocktail: ΦCP−p−KP−23240 + ΦCP−p−KP−21067	71−year−old male on peritoneal dialysis, refractory peritonitis (14−d IP antibiotics failed)	Intraperitoneal	Peritonitis resolved; discharged, healthy at 1−month follow−up	2026 (Yang et al., 2026)
Cocktail: ΦJD902 + ΦJD905 (first); Cocktail: ΦJD905 + ΦJD907 + ΦJD908 (combined with antibiotics) (second)	66-year-old patient, MDR *K. pneumoniae* UTI	Irrigated simultaneously via kidney and bladder	Discharged and did not recur after two months of follow−up	2023 (Liang et al., 2023)
Single phage: Φ59	54-year-old patient, *K. pneumoniae* pulmonary infection	Nebulization	Symptoms of cough and expectoration improved; inflammatory reaction reduced	2023 (Liang et al., 2023)

## 8 Phage endolysins and their therapeutic potential against *K. pneumoniae*

Phage endolysins play a critical role in the phage lytic cycle by specifically degrading the bacterial cell wall, thereby leading to rapid cell death (Phothichaisri et al., 2022). In recent years, significant progress has been made in research on phage endolysins for treating bacterial infections, progress underscored by the effective lytic activity of recombinantly expressed endolysins against *K. pneumoniae* (Ho et al., 2022). Similar to intact phages, endolysins possess high specificity, a property that enables them to target specific bacterial strains without harming other microorganisms or human cells (Fursov et al., 2020; Feng et al., 2021). For example, Vasina et al. demonstrated that phage endolysins do not affect the mouse gut microbiota, exhibit no cytotoxicity toward human cells, and do not induce short-term resistance *in vivo* (Vasina et al., 2021).

Structurally, phage endolysins typically consist of two modular domains: an enzymatically active domain (EAD) and a cell wall-binding domain (CBD). The EAD is responsible for degrading the bacterial cell wall by cleaving specific chemical bonds within the peptidoglycan layer, whereas the CBD interacts with bacterial surface polysaccharides, thereby influencing the specificity and efficacy of the endolysin (Zhang et al., 2025). For instance, the N-terminal EAD of CD16/50L exhibits potent peptidoglycan hydrolase activity, which enables the lysis of *Clostridioides difficile* cells. Mutants lacking this domain or harboring dimerization defects (e.g., W257A) demonstrate altered cell lytic activity, with effects varying in a strain-specific manner. Additionally, this domain facilitates the anchoring of endolysins to bacterial lysate residues, a function that prevents their diffusion and mitigates unnecessary lysis of adjacent, uninfected cells, thus promoting successful phage infection of new hosts (Phothichaisri et al., 2022). Similar principles apply to endolysins targeting *K. pneumoniae*. The endolysin LysSAP26 contains a CHAP−type EAD and a CBD domain, and this recombinant protein inhibits the growth of CRKP clinical isolates with an MIC value of 40 µg/mL (Kim et al., 2020a). Another endolysin is LysKP213 derived from phage KP2025, which contains a T4−like_lys EAD domain. When combined with polymyxin B or fused with the antimicrobial peptide cecropin A (CecA), it exhibits enhanced antibacterial activity against *K. pneumoniae* both *in vitro* and *in vivo* (Chu et al., 2024). Beyond EAD catalysis, the modular architecture of endolysins enables rational engineering. High−throughput screening of 940 engineered lysin variants targeting *K. pneumoniae* revealed that specific EADs are significantly overrepresented among top hits. Configurations such as (OMP–linker–CBD–EAD) or (CBD–EAD–linker–OMP) yield the most active variants. These findings further underscore the functional importance of EAD modules (Duyvejonck et al., 2021).

Phage endolysins exhibit broad-spectrum antibacterial activity against both Gram-positive and Gram-negative bacteria, showing significant potential against MDR strains. The efficacy of phage endolysins against MDR pathogens is well-documented by several studies. For instance, LysSAP26 and PlyKp104 show activity against ESKAPE pathogens, while LysSS is effective against MDR Gram-negative bacteria such as *Acinetobacter baumannii* (*A. baumannii*) and *K. pneumoniae* (Kim et al., 2020a; Kim et al., 2020b; Euler et al., 2023). Some endolysins, like XFII, possess a broader spectrum, lysing both Gram-negative bacteria and *Staphylococcus aureus* (*S. aureus*) (Zhang et al., 2022). Beyond direct bactericidal effects, LysCA and LysG24 inhibit *K. pneumoniae* and ameliorate pulmonary inflammation in animal models (Murray et al., 2021). However, the protective outer membrane of Gram-negative bacteria inherently reduces the activity and accessibility of endolysins (Kim et al., 2020b; Lim et al., 2022). To overcome this barrier, current strategies are twofold. On one hand, outer membrane permeabilizers (e.g., EDTA, chloroform, Triton X-100) or synergistic antimicrobials (e.g., colistin, polymyxins) are used to facilitate endolysin entry. On the other hand, recombinant endolysins are directly engineered to optimize their efficacy (Guo et al., 2017; Jiang et al., 2021; Yuan et al., 2021; Zaki et al., 2023).

Furthermore, phage endolysins demonstrate notable antibiofilm activity. Specific endolysins such as CF-301, P128, PlyF307, and LysSYL can effectively clear single-species or mixed-species biofilms (Liu et al., 2023; Liu et al., 2024). LysCP28, for instance, both prevents and removes Clostridium perfringens biofilms, whose activity can be further enhanced through genetic engineering or combination with other drugs (Liu et al., 2023; Lu et al., 2023). The applications of phage endolysins have extended to the food industry (controlling foodborne pathogens) and clinical settings (treating drug-resistant bacterial infections), making them promising alternative antimicrobials in the post-antibiotic era (Oechslin et al., 2022; Stevens et al., 2023). Applications of phage endolysins against *K. pneumoniae* are summarized in **Table 5**.

**Table 5:** Application efficacy and combined medication of phage endolysins in *K. pneumoniae*.

Endolysin name	Results: effect of use alone	Combination of reagents		Animal model test	
		Reagent	Effect of combined use	Animal infection model	Results/references
LysG24 and LysCA	Moderate antibacterial activity	EDTA	Significant enhancement of antibacterial activity	Mouse (pneumonia model)	Reduces pulmonary inflammation; Lowers bacterial load; Non-toxic in mice (Lu et al., 2022)
LysKP213	Optimal MIC: 21 μg/mL; High thermostability	Polymyxin B	Two-fold reduction in the MIC of polymyxin B	*G. mellonella*	Enhanced survival in larvae: Combination treatment > monotherapy (Chu et al., 2024)
LysECD7	Planktonic Killing: 99% in 5 min; Biofilm Disruption: Up to 77.6%	LysECD7-SMAP	Enhanced outer membrane permeability	Mouse (*Klebsiella* sepsis model)	Survival rate: 80.0% (2.5 mg/mL); 66.7% (5 mg/mL) Lung bacterial load: -2.2 log_10_ CFU/g (Vasina et al., 2024)

## 9 Phage resistance in *K. pneumoniae*: mechanisms, fitness costs and counterstrategies

The therapeutic efficacy of phages is challenged by the rapid emergence of resistant bacteria. *K. pneumoniae* has evolved multiple defense mechanisms to counteract phage predation. Understanding these mechanisms is essential for optimizing phage therapy.

### 9.1 Surface receptor modification

The most common resistance mechanism is inhibition of phage adsorption. This is achieved through mutations in genes encoding surface receptors such as CPS or LPS. For example, CRKP develops resistance by modifying CPS biosynthesis genes (Yu et al., 2025). In the K54-type hypervirulent *K. pneumoniae* strain SCNJ1, 97.3% of phage resistant clones carried CPS mutations with *wcaJ* as a hotspot (Fu et al., 2025a). In addition to genetic mutations, some strains downregulate capsule biosynthesis without any genetic changes, representing a reversible non-mutational mechanism (Mora-Quilis et al., 2025). Notably, phages can counter these bacterial defenses by mutating their own receptor binding proteins. For instance, phage ZX1Δint mutated its ORF59 protein to recognize an alternative binding site on the LPS O−antigen, thereby enabling reinfection of previously resistant strains (Li et al., 2025).

### 9.2 Nucleic acid degradation systems

Beyond surface defenses, *K. pneumoniae* employs multiple nucleic acid degradation systems to neutralize invading phages. The restriction−modification (R−M) system is a ubiquitous defense mechanism. Methyltransferases modify the bacterium’s own DNA while restriction endonucleases selectively degrade unmethylated foreign DNA (Yu et al., 2025). In hypervirulent strains, two distinct R−M systems have been identified: the Type IV restriction system McrBC and a unique Type I R−M system (Oo et al., 2025). Another well−characterized system is CRISPR−Cas, which provides sequence specific immunity by integrating short phage DNA fragments as spacers into the bacterial genome (Rendueles et al., 2023). Additional defense modules include BREX, DISARM and CBASS (Goldfarb et al., 2015; Ofir et al., 2018). These systems function by blocking phage DNA replication or cleaving incoming DNA. Many of these defense systems are not randomly distributed. Instead, they are often clustered together in specific chromosomal loci known as defense islands. Beyond genomic clustering, the expression of these defense systems is also subject to regulatory control. The quorum sensing (QS) system has been shown to play a role in this process. Inhibition of QS by cinnamaldehyde reduced phage resistance in *K. pneumoniae* and enabled a previously resistant strain to be infected by phage (Barrio-Pujante et al., 2024).

### 9.3 Abortive infection

A more radical resistance strategy is abortive infection (Abi). Unlike adsorption blocking or DNA degradation, Abi does not prevent phage entry. Instead, it triggers programmed bacterial cell death upon phage invasion. By sacrificing the infected cell, Abi systems stop phage replication and prevent the spread of progeny virions, thereby protecting the surrounding bacterial population. Activation of toxin−antitoxin modules is a key mediator of this response. This strategy is particularly common in hypervirulent *K. pneumoniae* strains and often coexists with surface receptor modifications to form a robust multi−layered defense. For example, ST11 CRKP commonly combines CPS biosynthesis mutations with TA mediated abortive infection (Yu et al., 2025).

### 9.4 Phage inducible chromosomal islands

In addition to the above mechanisms, phage infection can induce the excision and replication of phage inducible chromosomal islands (PICIs) from the bacterial chromosome. PICIs are mobile genetic elements that parasitize phages. Upon induction, they hijack phage structural proteins to assemble their own infectious particles. This molecular piracy sequesters essential phage structural components and thus reduces the production of functional phage progeny. PICIs also lower the frequency of lysogenization after temperate phage infection. This resistance mechanism has been identified in a substantial proportion of MDR *K. pneumoniae* isolates, underscoring its clinical relevance (Ibarra-Chávez et al., 2022).

### 9.5 Fitness costs

Although phage resistance provides a survival advantage under phage pressure, it frequently comes with fitness costs. These costs include reduced capsule production, attenuated virulence and restored susceptibility to clinically relevant antibiotics (Yu et al., 2025). For instance, in K2 capsule type hvKP infected by K2 specific phage ΦFK1979, resistant mutants showed decreased capsule expression and lower virulence (Tang et al., 2023). Similarly, in the K54-type hypervirulent *K. pneumoniae* strain SCNJ1, all CPS related mutants exhibited compromised fitness in mice, whereas a non−CPS mutant carrying a *bglA* mutation retained full fitness (Fu et al., 2025a). In a separate study using CRKP isolate KLEPN 2092, Yu et al. found that phage resistant mutants derived from this strain were significantly more susceptible to imipenem and meropenem than the parental strain. This finding further supports the trade off between phage resistance and antibiotic sensitivity (Yu et al., 2025). Moreover, stepwise resistance can alter antibiotic susceptibility in complex ways. Deletion of *ugd*/*wbgU* genes affected lipid A modification and changed receptor accessibility for phage P40 (Yin et al., 2026). Disruption of *rpoN* increased susceptibility to colistin, while *mutS* or *mutL* disruptions caused increased resistance to rifampicin and colistin (Nang et al., 2024).

### 9.6 Coevolution and counterstrategies

Despite the inevitability of phage resistance, phages can maintain infectivity through coevolution with their hosts. This antagonistic coevolution drives microbial community ecology (Koskella and Brockhurst, 2014). Bacteria have evolved defense systems such as BREX and DISARM, while phages have developed counterstrategies including anti−CRISPR proteins (Maxwell, 2016). To overcome host tolerance, researchers have proposed “phage training”. This approach harnesses the evolutionary potential of phages to improve infectivity and delay the emergence of resistance (Borin et al., 2021). Both evolutionary and coevolutionary “phage training” models have been shown to improve bacterial suppression. Notably, coevolutionary training selects for resistant bacteria with lower resistance levels compared to evolutionary training (Ngiam et al., 2024). Using dual receptor phages or targeting conserved surface structures may further reduce resistance evolution (Borges, 2021).

The diverse resistance mechanisms and their associated fitness costs provide a strong rationale for combination therapy. By combining phages with antibiotics, antimicrobial peptides or other agents, we can exploit the vulnerabilities of resistant mutants, suppress resistance emergence and enhance treatment efficacy.

## 10 Combination therapy

The diversity of phage resistance mechanisms and their associated fitness costs provides a strong rationale for combination therapy. Combining phages with antibiotics, antimicrobial peptides or other agents can exploit the vulnerabilities of resistant mutants. This approach suppresses resistance emergence and enhances treatment efficacy.

### 10.1 Phage cocktails

Phage cocktails employ multiple antibacterial mechanisms. They exhibit synergistic activity by targeting different bacterial sites or receptors, which broadens their antibacterial spectrum to include more species and resistant strains. This multi-target strategy, in turn, reduces the development of bacterial resistance and suppresses the emergence of phage-resistant mutants and biofilm formation (Han et al., 2023). The inherent differences in the mechanisms of action among the constituent phages in a cocktail mean that bacteria rarely develop simultaneous resistance to all components, a critical feature that lays the foundation for their therapeutic application.

Currently, personalized phage therapy has been successfully applied in Europe and the United States (Patil et al., 2021). Multiple studies have corroborated the characteristics and efficacy of phage cocktails in treating bacterial infections. For instance, phage cocktails have been shown to effectively treat *K. pneumoniae*-induced sepsis and improve the survival rate of *G. mellonella* infected with *K. pneumoniae* and *Klebsiella oxytoca* without toxicity (Singh et al., 2022; Kelly and Jameson, 2024). Chen et al. found that a phage cocktail (ΦK2044 + ΦKR1 + ΦKR8) effectively killed *K. pneumoniae* in biofilms and prevented biofilm formation. Furthermore, this cocktail delayed the emergence of phage resistance and improved the 7-day survival rate in both *G. mellonella* and mouse models, which demonstrates its therapeutic potential for applications such as catheter-associated biofilm infections (Chen et al., 2024). Similarly, the work of Singh et al. provides a clear demonstration that phage cocktails can achieve complete protection in mice during the fatal stage of acute sepsis while entirely preventing the emergence of resistant mutants (Singh et al., 2022).

Nevertheless, the antibacterial efficacy of phage cocktails can be influenced by factors such as phage selection, dose, and the specific infection model. Furthermore, they have the potential to disrupt the normal beneficial microbiota (Patil et al., 2021). Future research should therefore focus on further optimizing their formulation and application methods to enhance both antibacterial efficacy and clinical value. Selected clinical trials of phage cocktails are summarized in **Table 6**.

**Table 6:** Clinical trials of phage cocktails.

Phage cocktail	Targeted bacteria	Targeted diseases	Research (clinical trial number)	Result	Year of completion/ references
BX002-A	*K. pneumoniae*	Inflammatory Bowel Disease	Phase 1 study (NCT04737876)	Safe and well-tolerated; No serious adverse events; 10¹^0^ PFU to gastrointestinal tract achieved	2020 (Yang et al., 2023)
PYO	Enterococci, *E. coli*, *Proteus mirabilis*, *P. aeruginosa*, Staphylococci, Streptococci	Urinary tract infection	Phase 2/3 study (NCT03140085)	Efficacy and safety are non-inferior to standard-of-care antibiotics	2018 (Leitner et al., 2021)
AB-SA01	*S. aureus*	Wound	Phase 1 study (NCT02757755)	Well tolerated	2016 (Ooi et al., 2019)
WPP-201	*P. aeruginosa*, *S. aureus*, *E. coli*	Venous leg ulcers	Phase 1 study (NCT01818206, NCT00663091)	Favorable safety profile with no adverse events reported	2012 (Rhoads et al., 2009)

### 10.2 Combined antibacterial therapy with phages and antibiotics

Studies demonstrate that phage-antibiotic combinations generate synergistic effects that significantly enhance antibacterial efficacy. The underlying mechanism involves complementary functions where phages facilitate antibiotic penetration into bacterial cells, thereby improving bactericidal efficiency. Concurrently, antibiotics suppress the development of phage resistance in bacteria, which collectively enhances the overall antibacterial effect (Chang et al., 2022).

Multiple investigations support this finding. The combination of phage LAPAZ with meropenem exhibited synergistic antibacterial activity against *K. pneumoniae*, achieving complete bacterial eradication without phage resistance emergence (Ziller et al., 2024). In another study, Wang et al. demonstrated that combined treatment with *K. pneumoniae* phage P-KP2 and gentamicin cured a mouse model of acute pneumonia within six days (Wang et al., 2021). Eskenazi et al. further reported that phage M1 combined with ceftazidime/avibactam or meropenem completely cleared infections caused by pandrug-resistant *K. pneumoniae* (Eskenazi et al., 2022). Additionally, Nir-Paz et al. successfully treated polymicrobial bone infections using phage combinations with meropenem or colistin (Nir-Paz et al., 2019).

This combination strategy also broadens the antibacterial spectrum, thereby inhibiting a wider range of bacterial strains and improving overall therapeutic efficacy. Furthermore, it enables antibiotic dosage reduction, which lowers resistance development risk and provides significant potential for managing drug-resistant infections.

### 10.3 Combined antibacterial therapy with AMPs and phage endolysins

AMPs represent a class of small peptides, typically comprising 10–50 amino acid residues, that function as key effectors in innate immunity. These molecules serve as the first line of defense against pathogenic infections and demonstrate efficacy against diverse pathogens, including bacteria, viruses, fungi, and parasites (Rodríguez et al., 2021). **Table 7** provides an overview of selected AMPs. AMPs exhibit considerable clinical potential, particularly for the topical management of MDR and biofilm-associated infections. Cardoso et al. documented that Pa-MAP1.9 effectively inhibits *K. pneumoniae* ATCC biofilms through reduction of biofilm volume and height (Cardoso et al., 2016). Complementing this finding, Zhou et al. confirmed that AMP A20L displays potent antibacterial and antibiofilm activity in laboratory models while demonstrating anti-infective efficacy in animal studies (Zhou et al., 2024).

**Table 7:** Action targets, resistance mechanisms, and key advantages of representative AMPs.

AMPs	Target site of action	Drug resistance mechanism	Key advantages	Reference
LTX-315	Phosphatidylglycerol	Membrane disruption	Broad-spectrum activity; Low toxicity	(Tang et al., 2025)
PepW	Capsular polysaccharide, Cell membrane	Capsular barrier	Capsular barrier penetration	(Fleeman et al., 2020)
DGL13K	Cell membrane	Regulatory system activation	Low resistance induction; Biofilm inhibition and disruption	(Gorr et al., 2022)
K11	Cell membrane	Membrane alteration	Synergy with conventional antibiotics; Anti-biofilm activity	(Chatupheeraphat et al., 2023)
WAM-1	Cell membrane	Dual resistance mechanism	Potent bactericidal effect against CRKP	(Zhang et al., 2022)
PvAMP66	Cell membrane	Efflux-mediated resistance	Synergy with gentamicin	(Salinas-Restrepo et al., 2023)

The combination of AMPs with phage endolysins generates synergistic antibacterial effects that broaden the antimicrobial spectrum. This approach simultaneously reduces resistance development and enhances biofilm clearance capacity. Experimental evidence from both *in vitro* and animal studies confirms effectiveness against multiple bacterial pathogens, including *P. aeruginosa*, *Enterococcus faecalis*, *A. baumannii*, and *K. pneumoniae* (Hesse et al., 2020). Notable examples illustrate this synergy. The combination of Cecropin A with phage endolysin LysGH15 significantly enhances bactericidal activity against methicillin-resistant *S. aureus* (MRSA) and shows promising efficacy in murine infection models. Further advancing this approach, Islam et al. demonstrated that fusing Cecropin A’s N-terminus with Ab endolysin substantially improves bactericidal activity against MDR *A. baumannii* clinical isolates (Islam et al., 2022).

## 11 Conclusion and outlook

*K. pneumoniae* continues to pose a critical threat to global public health, driven by the rapid spread of MDR and extensively drug-resistant (XDR) strains. The diminishing efficacy of conventional antibiotics has accelerated the search for alternative therapeutic strategies. Among them, phage therapy and phage-derived enzymes (especially endolysins and polysaccharide depolymerases) represent promising therapeutic candidates. These agents offer distinct advantages, including high target specificity, biofilm disrupting capacity, and low cross resistance with antibiotics. Preclinical studies in animal models have consistently demonstrated their ability to reduce bacterial loads and improve survival across various infection types, reinforcing their therapeutic potential.

Despite these advances, the clinical translation of phage-based and endolysin-based therapies for *K. pneumoniae* faces several critical challenges. The narrow host range of many *K. pneumoniae* specific phages limits their applicability against diverse capsular serotypes and clonal lineages prevalent in clinical settings. Moreover, phage resistance emerges rapidly via surface receptor modifications (e.g., LPS, CPS), phage DNA entry inhibition, or the activation of bacterial defense systems (e.g., CRISPR-Cas, BREX), which can impair therapeutic efficacy. For endolysins, the Gram-negative outer membrane remains a substantial biophysical barrier, often requiring membrane permeabilizers or protein engineering, which introduce additional complexities in formulation and safety. Knowledge gaps also persist regarding the *in vivo* pharmacokinetics, immune clearance, and long term effects on the commensal microbiota. Furthermore, there is a lack of standardized protocols for phage isolation, characterization and cocktail development, and regulatory uncertainties further impede clinical translation.

**Figure 4:**
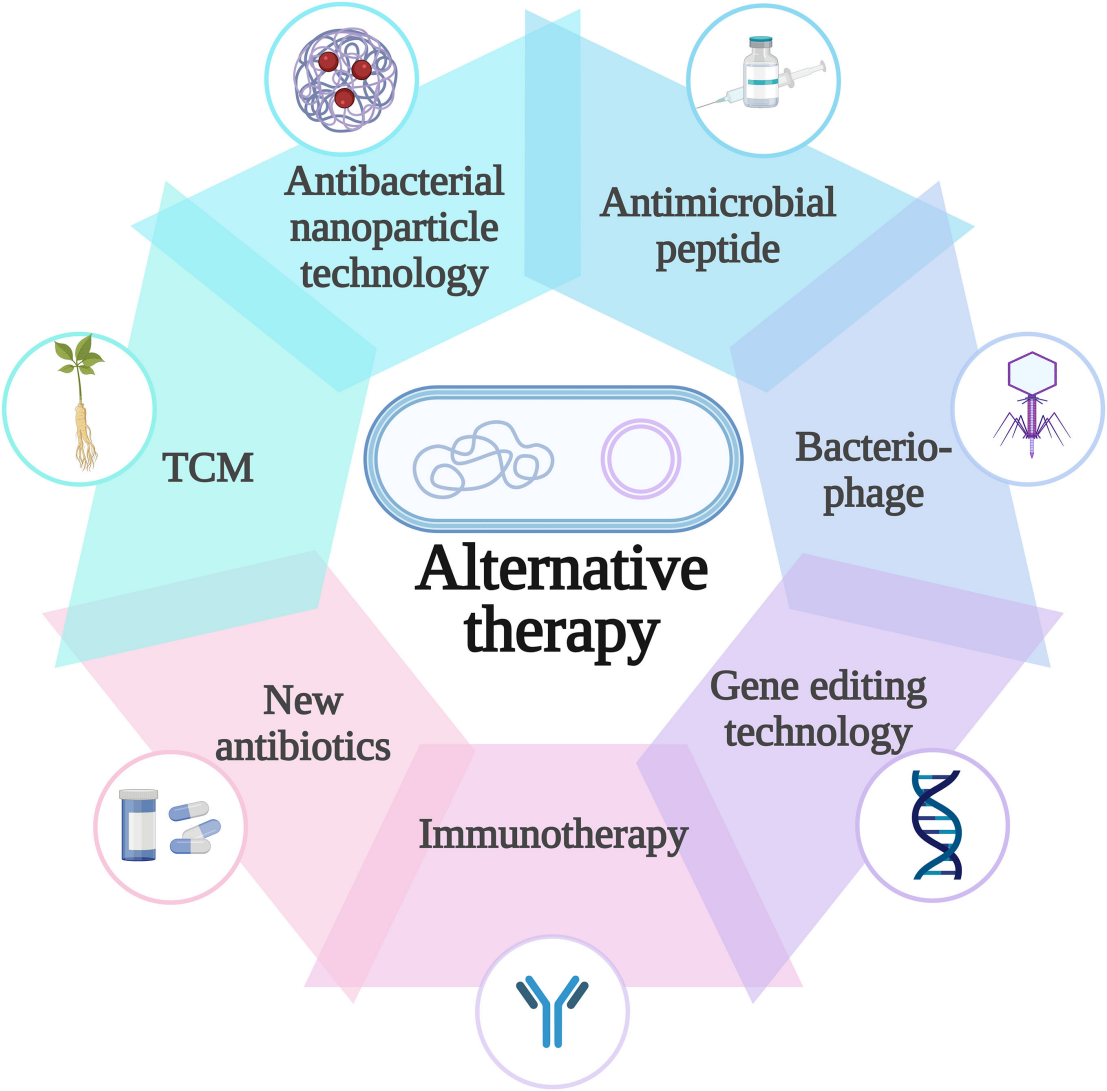
Alternative therapies to traditional antibiotics: (1) new antibiotics: developed through bacterial genomics, they overcome porin-mediated resistance and provide non-β-lactam options. (2) AMPs: Kill bacteria via membrane disruption and immunomodulation, and can enhance the efficacy of conventional antibiotics. (3) Bacteriophage: Specifically infect and lyse bacteria, penetrate deep-seated tissues, and degrade biofilms. (4) TCM: Components such as baicalein reduce resistance and inhibit biofilm formation with a low risk of inducing further resistance. (5) Immunotherapy: Vaccines and antibodies help prevent infection and mitigate resistance, though immunogenicity challenges remain. (6) Antibacterial nanoparticles: Disrupt bacterial membranes and induce ROS. Their combination with antibiotics or photothermal therapy enhances efficacy. (7) Antisense oligonucleotides and gene editing: Combat resistance by suppressing resistance gene expression or precisely removing resistance genes at the genetic level.

Addressing these hurdles requires a multifaceted research agenda. First, structural and molecular studies should elucidate *K. pneumoniae*-phage interactions to guide rational engineering of phages and endolysins with expanded host tropism and enhanced outer membrane activity. Strategies such as modular optimization of endolysin domains, refinement of “phage training” protocols, and exploration of dual receptor phages targeting conserved surface structures warrant particular attention. Second, preclinical investigations must transition toward large animal models (e.g., rabbits, swine) to optimize dosing regimens, delivery routes, and formulations that improve *in vivo* stability and bioavailability. Third, translational efforts should prioritize standardized platforms for phage discovery and rational design of serotypically diverse phage cocktails. Well-designed Phase 2/3 randomized controlled trials are urgently needed to evaluate phage cocktails and phage antibiotic combinations for severe infections such as carbapenem-resistant *K. pneumoniae* pneumonia and bacteremia. Fourth, interdisciplinary collaboration should explore integrating phage-based therapies with new antibiotics, host directed immunotherapies, antibacterial nanoparticles, AMPs and Chinese medicine (TCM) to establish multimodal regimens that suppress resistance evolution, as illustrated in **Figure 4**. Exploring the pleiotropic effects of phage resistance (e.g., enhanced antibiotic sensitivity) also provides opportunities to develop synergistic therapeutic regimens.

In summary, phage-based and endolysin-based therapies should be regarded not as standalone alternatives, but as integral components of a broader antimicrobial armamentarium against drug resistant *K. pneumoniae*. Realizing their clinical potential will require sustained investment in basic and translational research, international cooperation to harmonize development and regulatory frameworks, and their thoughtful incorporation into antimicrobial stewardship initiatives. As mechanistic understanding deepens and translational pipelines mature, these modalities hold the potential to transform therapeutic options for patients with limited alternatives. ^*^Created with BioGDP.com (Jiang et al., 2025).
